# Role of protein kinase R in the killing of Leishmania major by macrophages in response to neutrophil elastase and TLR4 v*ia* TNFα and IFNβ

**DOI:** 10.1096/fj.13-245126

**Published:** 2014-07

**Authors:** Marilia S. Faria, Tereza C. Calegari-Silva, Aislan de Carvalho Vivarini, Jeremy C. Mottram, Ulisses Gazos Lopes, Ana Paula C. A. Lima

**Affiliations:** * Instituto de Biofisica Carlos Chagas Filho, Universidade Federal do Rio de Janeiro, Rio de Janeiro, Brazil; and; † The Wellcome Trust Centre for Molecular Parasitology, Institute of Infection, Immunity, and Inflammation, College of Medical, Veterinary, and Life Sciences, University of Glasgow, Glasgow, UK

**Keywords:** ecotin, Toll, interferon, ISP2

## Abstract

In cutaneous leishmaniasis, Leishmania amazonensis activates macrophage double-stranded, RNA-activated protein kinase R (PKR) to promote parasite growth. In our study, Leishmania major grew normally in RAW cells, RAW-expressing dominant-negative PKR (PKR-DN) cells, and macrophages of PKR-knockout mice, revealing that PKR is dispensable for L. major growth in macrophages. PKR activation in infected macrophages with poly I:C resulted in parasite death. Fifty percent of L. major-knockout lines for the ecotin-like serine peptidase inhibitor (ISP2; Δ*isp*2/*isp*3), an inhibitor of neutrophil elastase (NE), died in RAW cells or macrophages from 129Sv mice, as a result of PKR activation. Inhibition of PKR or NE or neutralization of Toll-like receptor 4 or 2(TLR4 or TLR2) prevented the death of Δ*isp*2/*isp*3. Δ*isp*2/*isp*3 grew normally in RAW-PKR-DN cells or macrophages from 129Sv *pkr*^−/−^, *tlr2*^−/−^, *trif*^−/−^, and *myd88*^−/−^ mice, associating NE activity, PKR, and TLR responses with parasite death. Δ*isp*2/*isp*3 increased the expression of mRNA for TNF-α by 2-fold and of interferon β (IFNβ) in a PKR-dependent manner. Antibodies to TNF-α reversed the 95% killing by Δ*isp*2/*isp*3, whereas they grew normally in macrophages from IFN receptor–knockout mice. We propose that ISP2 prevents the activation of PKR *via* an NE-TLR4-TLR2 axis to control innate responses that contribute to the killing of L. major.—Faria, M. S., Calegari-Silva, T. C., de Carvalho Vivarini, A., Mottram, J. C., Lopes, U. G., Lima, A. P. C. A. Role of protein kinase R in the killing of Leishmania major by macrophages in response to neutrophil elastase and TLR4 *via* TNFα and IFNβ.

Double-stranded RNA (dsRNA)-activated serine/threonine kinase, protein kinase R (PKR), is a well-characterized component of antiviral response. Classically, PKR is autophosphorylated on binding to dsRNA and phosphorylates the subunit of eIF-2a, resulting in rapid inhibition of translation (1). Lately, there is growing evidence of a role for PKR in response to bacterial lipopolysaccharide (LPS), through the activation of NF-κB and regulation of the expression of TNF-α and IL-6 (2, 3). In alveolar macrophages, both Toll-like receptor 2 (TLR2; Pam3CSK4) and TLR4 (LPS) ligands induce rapid phosphorylation of PKR that is strictly dependent on functional TLR (4).

Leishmania sp. are flagellated protozoan parasites that cause leishmaniasis worldwide (reviewed in ref. 5). Different species cause diverse clinical symptoms, varying from localized skin ulcerations to the visceral form, which can be lethal if untreated (6). Genome-wide analyses have revealed high gene conservation between different Leishmania species, showing that there are very few species-specific genes (7). Therefore, the regulation of gene expression and the molecular interactions in the parasite–host context seem to be crucial in determining the outcome of the disease. Infection with L. amazonensis can provoke diffuse cutaneous lesions, whereas infection with L. major leads to localized self-healing lesions. The infective form of the parasite (metacyclic promastigote) is injected in the dermis of the mammalian host by sandflies, where it is internalized by phagocytes. The parasite establishes infection in macrophages and multiplies as amastigotes in a parasitophorous vacuole, where they are protected from microbicidal components produced by those phagocytes. The mechanisms that enable parasite survival and growth in macrophages are complex and often include significant down-regulation of innate inflammatory mediators by the host cell (reviewed in ref. 8).

L. major has 3 genes, inhibitor of serine peptidases 1–3 (*ISP1*, *ISP2*, and *ISP3*), that encode proteins with similarity to the bacterial serine peptidase inhibitor ecotin (9). ISP2 is functional and inhibits neutrophil elastase (NE), found at the surface of macrophages (10). The use of L. major lines deficient in *ISP2* and *ISP3* (Δ*isp*2/*isp*3) revealed that inhibition of NE by ISP2 is necessary to prevent the triggering of TLR4 responses at the moment of phagocytosis. In the absence of ISP2, activation of the NE-TLR4 pathway results in the killing of a proportion of intracellular parasites and delayed growth of the parasites that remain (10). The nature of the NE-TLR4 downstream responses triggered at infection is unknown.

Recently, PKR activation has been implicated in the successful infection of macrophages by L. amazonensis. Infection induces PKR activation and expression that correlates with increased expression of IL-10 and is necessary for optimal parasite growth (11). Remarkably, the activation of NF-κB (p65/p50) induced by PKR on macrophage treatment with polyinosinic:polycytidylic acid [poly(I:C)] is altered when the cells are coinfected with L. amazonensis. The parasite induces a shift toward the repressor p50/p50 NF-κB form and cleavage of the p65 subunit. In contrast, L. major is incapable of altering NK-kB proteins, preserving poly(I:C)-induced p65/p50 activation. This finding leads to the hypothesis that PKR activation by L. major could exert a negative effect on parasite growth related to the activation of inflammatory genes by NF-κB.

A follow-up study showed that PKR activation during L. amazonensis infection induces type 1 interferon β (IFNβ), which, in turn, up-regulates the expression of superoxide dismutase 1 (SOD1), possibly reducing the levels of reactive oxygen species (ROS) (12). All responses related to PKR activation in L. amazonensis-infected macrophages depended on TLR2, suggesting that TLR-mediated responses are favorable to the growth of L. amazonensis through a PKR-IFNβ-SOD1 axis.

Since deficiency in *ISP*2 in L. major leads to the activation of TLR4, we asked whether PKR activation could occur downstream of TLR4, hence up-regulating inflammatory responses that could contribute to parasite death. In this study, we showed that, in the absence of ISP2, NE-TLR4 responses induced by L. major depend on functional PKR, resulting in the modulation of a PKR-TNF-α-IFNβ axis that contributes to parasite killing.

## MATERIALS AND METHODS

### Cell lines

RAW 264.7 cells were cultured in DMEM with high glucose and l-glutamine (Invitrogen, Carlsbad, CA, USA) and 10% heat-inactivated fetal calf serum (FCS; Cultilab, Campinas, Brazil), at 37°C in a 5% CO_2_ humidified atmosphere. RAW 264.7 cells expressing catalytically inactive dominant-negative PKR-K296R (RAW-DN-PKR cells) or empty pRcCMV vector (RAW-bla cells) were generated (13). Bone marrow–derived macrophages from 129Sv mice, *TRIF*^−/−^ mice [donated by Dr. S. Oliveira, Universidade Federal de Minas Gerais (UFMG), Belo Horizonte, Brazil], *MyD88*^−/−^ mice[donated by Dr. M. Bellio, Universidade Federal de Rio de Janeiro (UFRJ)], or *pkr*^−/−^ mice were obtained by washing bone marrow in cold RPMI, followed by culturing in RPMI 1640 (Invitrogen) supplemented with 20% FCS and 30% conditioned medium of the L929 cell line, for 10 d. The nonadherent cells were removed by washing, and the adherent cells were detached in cold RPMI, plated on glass coverslips, and cultured overnight in RPMI-FCS and 5% conditioned medium from L929 cells before the infection assays.

### Parasites

L. major Friedlin (MHOM/JL/80/Friedlin) were grown as promastigotes in hemoflagellate modified Eagle's medium (HOMEM) supplemented with 10% heat-inactivated FCS at 27°C (9). Parasite lines deficient in *ISP2* and *ISP3* (Δ*isp*2/*isp*3) and the reexpressing line (Δ*isp*2/*isp*3:*ISP*2/*ISP*3) were generated (9). Purified metacyclic promastigotes were obtained from stationary-phase cultures by washing and incubation with peanut lectin (Sigma-Aldrich, São Paulo, Brazil) at 50 μg/ml for 15 min, followed by centrifugation and recovery in the supernatant (14).

### Infection assays

All infections *in vitro* were performed with stationary-phase promastigotes at a 5:1 or 10:1 parasite:cell ratio, in triplicate. Macrophages or RAW cell lines were plated on glass coverslips and cultured in RPMI and 10% FCS overnight at 37°C. The cultures were washed and infected in DMEM supplemented with 0.1% bovine serum albumin (BSA) for 3 h at 37°C, followed by washing, fixation with 70% methanol, and Giemsa staining. The number of intracellular parasites was determined by counting ≥100 cells/replicate under a light microscope. Where indicated, the irreversible inhibitor of NE (NEi), *O*-methoxy-succinyl-alanyl-alanyl-prolyl-alanyl-chloromethylketone (OMeSuc-AAPV-CMK; 10 μM; Calbiochem, La Jolla, CA, USA), was added to the macrophages 5 min before addition of the parasites. Anti-mouse CD11b mAbs (clone M1/70), anti-mouse TLR4 neutralizing mAbs (clone MTS510), anti-mouse TLR2 (T2.5), or rat IgG2b (BD Biosciences, San Jose, CA, USA) was incubated at 10 or 5 μg/ml in DMEM/10% FCS for 30 min and removed by extensive washing before the addition of the parasites in DMEM-BSA. For the survival assays, the cells were infected in DMEM-BSA for 3 h, washed, and further cultured at 37°C in DMEM/10% FCS for 24 h. Where indicated, the cultures were washed and treated with 2 mM 2-aminopurine (2AP) for 1 h, followed by treatment with poly(I:C) at 25 μg/ml for 24 h, or with poly(I:C) alone. Peritoneal macrophages were elicited from C57B6, *TLR2*^*[*minus]/−^, 129Sv, or *IFN*γ*R*^−/−^ mice with thioglycolate and cultured overnight in RPMI/10% FCS at 37°C before infection.

### Immunofluorescence

Thioglycolate-recruited macrophages were cultured overnight in RPMI-FCS. The cells were infected in RPMI-BSA for 1 h, washed with PBS, and processed for immunofluorescence (10). Coverslips were incubated with anti-TLR2/CD282 (T2.5; eBioscience, San Diego, CA, USA) at 1:200 dilution for 2 h, followed by goat anti-mouse IgG-Cy3 (Jackson ImmunoResearch Laboratories, West Grove, PA, USA). The coverslips were subsequently incubated with rabbit polyclonal antibodies to TLR4 (ab 13556; Abcam, Cambridge, MA, USA) at 1:200 for 1 h, followed by mouse anti-rabbit IgG-Alexa 488; mounted on DAPCO/DAPI slides (Sigma-Aldrich); and observed in a confocal microscope (Zeiss, Thornwood, NY, USA) Each sample was sectioned from the bottom to top at 0.9 μm for stacks. In Supplemental Fig. S1, infected macrophages were incubated with anti-mouse activated caspase 3 (Abcam) at 1:100 overnight.

### Immunoblot analysis

RAW cells (4×10^6^) were infected in DMEM-FCS for the indicated times and washed in cold PBS, and nuclear extracts were prepared (12). Protein (20 μg) was separated on 10% SDS-polyacrylamide gels, and Western blot analysis was performed, with the anti-mouse p65 subunit of NF-κB (1:500; Santa Cruz Biotechnology, Santa Cruz, CA, USA), anti-mouse IRF3 (1:500), or anti-inducible nitric oxide synthase (iNOS; 1:200) (Santa Cruz Biotechnology). The loading controls were assessed with rabbit antibodies to lamin A/C (1:1000) overnight at 4°C or to β-tubulin (1:1000; Cell Signaling Technology, Danvers, MA, USA). Densitometries were determined by using ImageJ (U.S. National Institutes of Health, Bethesda, MD, USA) and normalized to the loading controls. Increases or reductions were then calculated in relation to nonstimulated controls, which were set at 1.0.

### ELISA and determination of nitrite concentrations

Cells (2×10^5^/well) were cultured overnight in RPMI-FCS, followed by replacement with fresh medium supplemented with recombinant IFNγ (100 ng/ml) for 3 h, and infected at a 10:1 parasite:cell ratio overnight at 37°C. The supernatants were collected, the parasites were removed by 5000 *g* centrifugation, and the cytokine concentration was determined by ELISA (BD Biosciences). Nitrite was measured with 50 μl of culture supernatants by the Griess method (15).

### Luciferase reporter assay

RAW cells (2×10^5^/well) were transfected with 1 μg of the plasmid 6KB (6 consensus binding sites for NF-κB; kindly provided by Dr. P. Bauerle, Ludwig-Maximilians-Universität, Munich, Germany; ref. 12). The cells were cotransfected with pRL-CMV (80 ng), which expresses Renilla constitutively (Promega, Fitchburg, WI, USA) for luciferase activity normalization. The cells were infected overnight in RPMI-FCS, washed with PBS, and lysed according to the dual-luciferase system protocol (Promega), and the lysates were analyzed in a TD-20/20 luminometer (Turner Designs, Sunnyvale, CA, USA).

### qPCR assays

Cells were infected for 4 h and washed, total RNA was extracted with the RNeasy minikit (Qiagen, Valencia, CA, USA), and cDNA was synthesized with 1 μg of RNA and the Improm Kit (Promega). Real-time PCR assays of first-strand cDNA were performed with Step One (Applied Biosystems, Foster City, CA, USA) and SYBR Green (Promega). The analyses were made using the Step One 2.0 software (Applied Biosystems). The primers used were: GAPDH (sense 5′-TGCACCACCACCTGCTTAGC-3′, antisense 5′-GGCATGGACTGTGGTCATGAG-3′); PKR (sense 5′-GACCTTCCTGACATGAAAGAA-3′, antisense 5′-AACTATTTTTGCTGTTCTCAGG-3′); iNOS (sense: 5′-CAGCTGGGCTGTACAAACCTT-3′, antisense: 5′-ATTGGAAGTGAAGCGTTTCG-3′); IFNβ (sense: 5′-TCCAAGAAAGGACGAACATTCG-3′, antisense: 5′-TGAGGACATCTCCCACGTCAA-3′); IL-10 (sense: 5′-CCCAGAAATCAAGGAGCATT-3′, antisense: 5′-TCACTCTTCACCTGCTCCAC-3′); and TNF-α (sense: 5′-GGTCCCCAAAGGGATGAGAAGTTC-3′, antisense: 5′-CCACTTGGTGGTTTGCTACGACG-3′).

### Mouse infections

Footpads of mice (5/group) were injected with 3 × 10^5^ purified metacyclic promastigotes. After 48 h, the popliteal lymph nodes and footpad tissue were collected and ruptured to homogeneity through a nylon membrane in 2 ml HOMEM. Cell homogenates (100 μl) were subjected to 2-fold serial dilutions in HOMEM-FCS in 48-well plates. The cells were cultivated at 27°C for 5–7 d, and the wells were inspected for parasite growth. The positive wells at the highest dilutions were used to estimate the number of parasites multiplied by the dilution factors.

### Statistical analyses

Statistical analyses were performed using the Prism program (GraphPad, San Diego, CA, USA) and 1- or 2-way ANOVA, with the Bonferroni *post hoc* test.

## RESULTS

**Figure 1.**
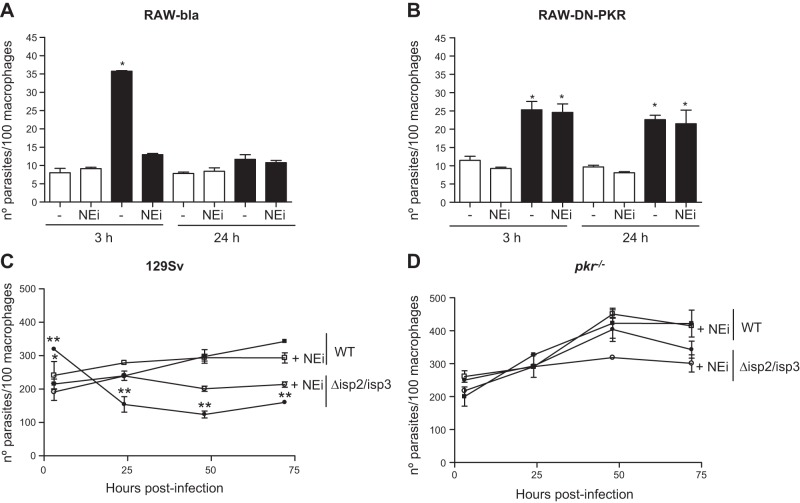
NE-dependent phagocytosis and killing of L. major Δ*isp*2/*isp*3 by macrophages requires PKR. *A*, *B*) RAW264.7 cell lines stably transfected with empty plasmid (RAW-Bla; *A*) or with the dominant-negative PKR construct (RAW-DN-PKR; *B*) were infected with L. major for 3 h, washed, and fixed (3 h) or cultured further overnight (24 h). *C*, *D*) Macrophages from 129Sv mice (*C*) or *pkr*^−/−^ mice (*D*) were infected for 3 h as just described, washed, and cultured for 48–72 h. Where indicated, the NEi OMeSuc-AAPV-CMK was added at 10 μM. Experiments were performed 3 independent times in triplicates. Graphs are representative of 1 experiment and indicate means ± sd of experiments performed in triplicate. Statistical analyses were performed using 1-way (*A*, *B*) or 2-way (*C*, *D*) ANOVA. Open bars: WT L. major; solid bars: Δ*isp*2/*isp*3 L. major. *P < 0.001, **P < 0.0002.

To address whether PKR influences the infection of macrophages by L. major, we initially used the RAW-DN-PKR cells as a tool. The uptake and survival of WT L. major was not affected in RAW-DN-PKR cells, suggesting that PKR does not influence the initial infection of macrophages by L. major (**Fig. 1** open bars). The uptake of Δ*isp*2/*isp*3 (Fig. 1, solid bars) by RAW-bla cells was higher than in the wild-type (WT) L. major (Fig. 1*A*). The number of intracellular Δ*isp*2/*isp*3 parasites decreased by ∼50% in RAW-bla cells after 24 h. In contrast, the uptake of Δ*isp*2/*isp*3 by the RAW-DN-PKR cells was lower than that by the RAW-bla cells, but still more efficient than the WT in the RAW-DN-PKR cells (Fig. 1*B*). Intracellular Δ*isp*2/*isp*3 parasites survived in those macrophages, indicating that functional PKR is necessary for the killing of L. major lacking ISP2 and ISP3. The enhanced entry and poor survival of Δ*isp*2/*isp*3 correlated with the activity of NE during phagocytosis, resulting in the mobilization of TLR4 (9). The addition of an NEi, OMeSuc-AAPV-CMK, reversed the increased uptake of the Δ*isp*2/*isp*3 parasites and their subsequent death in RAW-bla cells (Fig. 1*A*) but had no effect in RAW-DN-PKR cells (Fig. 1*B*). This observation suggests that the downstream effectors of unregulated NE activity during the phagocytosis of Δ*isp*2/*isp*3 converge with the activation of PKR. We analyzed next the kinetics of parasite intracellular growth in peritoneal macrophages derived from knockout mice lacking PKR (*pkr*^−/−^; Fig. 1*D*) or from the background strain of 129Sv mice (Fig. 1*C*). WT L. major developed normally in macrophages from the 129Sv (Fig. 1*C*) or *pkr*^−/−^ mice (Fig. 1*D*) revealing that, contrary to observations with L. amazonensis, PKR is not essential for the growth of L. major in macrophages.

In contrast, Δ*isp*2/*isp*3 parasites were internalized more efficiently than the WT by macrophages of 129Sv mice, but ∼50% were eliminated within 24 h (Fig. 1*C*). The remaining Δ*isp*2/*isp*3 parasites recovered the ability to multiply after 48 h. The addition of NEi reversed the increased internalization of the Δ*isp*2/*isp*3 and partially restored intracellular growth, at 24 h. We also observed a modest effect of NEi on the growth of WT parasites at 24 h. However, NEi seemed to affect the growth of intracellular WT or Δ*isp*2/*isp*3 parasites between 48 and 72 h in macrophages from the 129Sv mice. There was no difference in uptake, survival, or intracellular growth between WT and Δ*isp*2/*isp*3 up to 48 h in macrophages of the *pkr*^−/−^ mice (Fig. 1*D*). Infected macrophages were healthy at 72 h (Supplemental Fig. S1*A*), and no evidence of induction of apoptosis was detected, as assessed by labeling-activated caspase 3 (Supplemental Fig. S1*B*).

**Figure 2.**
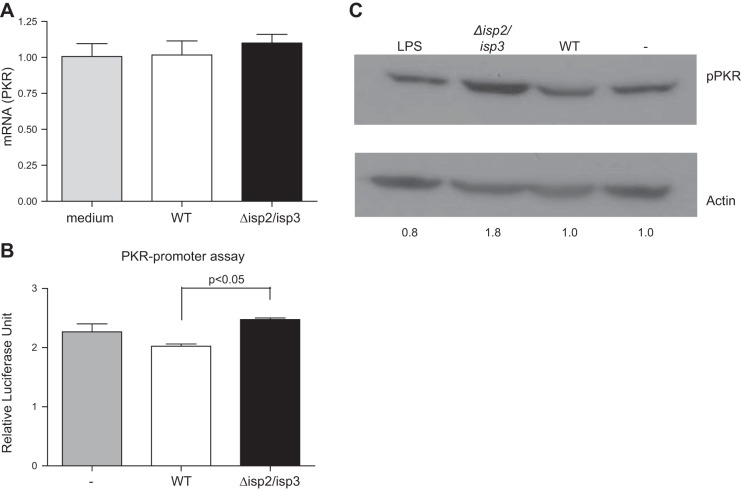
Infection of macrophages with L. major Δ*isp*2/*isp*3 promotes the activation of PKR. *A*) Real-time PCR for PKR. RAW264.7 cells were infected for 4 h and washed, and the cDNA samples were used as templates in qPCR. Noninfected cells were used as the control for PKR mRNA levels. Bars represent means ± sd of experiments performed 3 independent times. *B*) Assay of PKR promoter activation: RAW264.7 cells were transfected with the construct p503-WT, which contains the KCS and ISRE promoter elements upstream of the luciferase reporter gene, and infected for 24 h. Normalized lysates were tested for luciferase activity. Experiments were performed 2 independent times in triplicate. Bars are representative of 1 experiment and indicate means ± sd of experiments performed in triplicate. *C*) PKR phosphorylation. RAW264.7 cells were infected for 30 min, and the lysates were subjected to Western blot analysis, with anti-phospho-PKR and anti-actin antibodies as the loading control. Relative levels of phospho-PKR, calculated by densitometry, are indicated at bottom.

Next, we evaluated the phosphorylation/activation and expression of PKR. We did not detect alterations in the mRNA levels for PKR in RAW-bla cells infected with WT L. major or with Δ*isp*2/*isp*3 (**Fig. 2*A***), as compared to noninfected cells. Tests of PKR promoter activation were conducted using luciferase as a reporter gene, confirming negligible alterations in luciferase levels in macrophages infected with WT L. major (Fig. 2*B*). There was a modest increase in the luciferase activity of cells infected with Δ*isp*2/*isp*3 compared to WT, but this was not significantly higher than that of uninfected cells. PKR activation was then addressed by assessing the degree of PKR phosphorylation, revealing higher amounts of phospho-PKR in lysates of macrophages infected with Δ*isp*2/*isp*3, as compared to that in uninfected cells. In macrophages infected with WT L. major, the increase in phosphorylation was negligible, suggesting that Δ*isp*2/*isp*3 induces significant PKR activation on infection (Fig. 2*C*).

**Figure 3.**
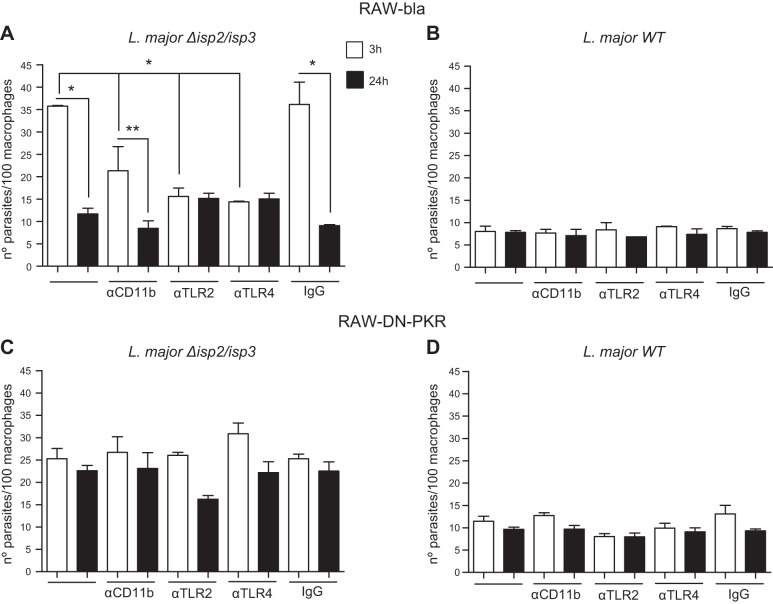
PKR-dependent modulation of Δ*isp*2/*isp*3 L. major survival requires TLR2, CD11b, and TLR4. RAW-Bla (*A*, *B*) or RAW-DN-PKR (*C*, *D*) cells were infected with Δ*isp*2/*isp*3 (*A*, *C*) or WT L. major (*B*, *D*) for 3 h, washed and fixed (3 h), or washed and cultured overnight (24 h). Where indicated, cultures were preincubated with 5 μg/ml of antibodies to CD11b, TLR4, TLR2, or control rat IgG2a for 30 min and washed before the addition of parasites. Experiments were performed 3 independent times. Graphs are representative of 1 experiment and indicate means ± sd. *P < 0.0001, **P < 0.05.

**Figure 4.**
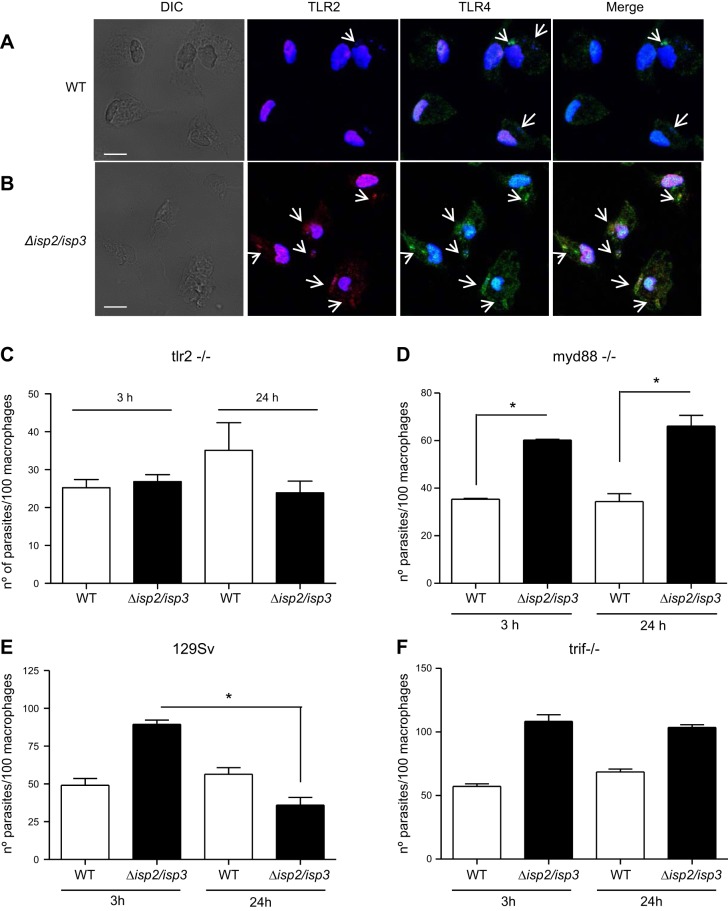
TLR2 and TLR4 are recruited to the site of entry of Δ*isp*2/*isp*3 L. major in macrophages, and parasite killing requires MyD88 and TRIF. *A*, *B*) Macrophages of C57B6 mice were infected with WT (*A*) or Δ*isp*2/*isp*3 L. major (*B*) for 1 h and processed for immunofluorescence, with anti-TLR2 (red) or anti-TLR4 (green). Samples were analyzed by confocal microscopy, sectioned from bottom to top at 0.9 μm, 8 times; images show section 3. Scale bars = 5 μm. Arrows indicate the parasite. *C–F*) Macrophages from TLR2-knockout mice (*C*), MyD88-knockout mice (*D*), 129Sv mice (*E*), or TRIF-knockout mice (*F*) were infected for 3 h, washed, and fixed or cultivated for a further 24 h. Experiments were performed 2 separate times. *P < 0.001.

Next, the cellular receptors involved in such processes were assessed by using neutralizing antibodies (**Fig. 3**). Preincubation of RAW-bla cells with IgG to TLR2, TLR4, or CD11b, but not with control IgG, prevented the enhanced phagocytosis and the elimination of intracellular Δ*isp*2/*isp*3 24 h after uptake (Fig. 3*A*). The observation that the antibodies to each individual receptor reduced the uptake of Δ*isp*2/*isp*3 to the same level suggests that the 3 receptors act conjointly to promote the enhanced phagocytosis of Δ*isp*2/*isp*3. Incubation with the antibodies did not affect the uptake or the survival of WT parasites in the RAW-bla (Fig. 3*B*) or RAW-DN-PKR (Fig. 3*D*) cells. In the RAW-DN-PKR cells, the levels of Δ*isp*2/*isp*3 uptake and their survival were unaffected by the antibodies to TLR2, TLR4, or CD11b, indicating that PKR activity is necessary to kill them and is conveyed through the above-mentioned receptors (Fig. 3*C*). The association of TLR2 and TLR4 with the uptake of L. major was further addressed using primary macrophages (**Fig. 4**). Confocal microscopy of macrophages infected with WT L. major showed colocalization of a few internalized parasites with TLR4 but no colocalization with TLR2 (Fig. 4*A*). In contrast, we observed marked colocalization between Δ*isp*2/*isp*3, TLR2, and TLR4, suggesting that both TLRs are brought together and internalized in the phagocytic vesicles with the mutant parasite (Fig. 4*B*). The involvement of TLR2 was confirmed by the infection of macrophages derived from *TLR*2-knockout mice (Fig. 4*C*). In those macrophages, Δ*isp*2/*isp*3 parasites were internalized at the same levels as WT, and the number of intracellular parasites was unchanged after 24 h. The infection of macrophages originating from mice defective in the adaptor proteins MyD88 (Fig. 4*D*) or TRIF (Fig. 4*F*) revealed that the killing of intracellular Δ*isp*2/*isp*3 is dependent on both adaptor proteins. Macrophages from control 129Sv mice displayed killing of ∼70% of intracellular Δ*isp*2/*isp*3 (Fig. 4*E*), but not of WT L. major.

**Figure 5.**
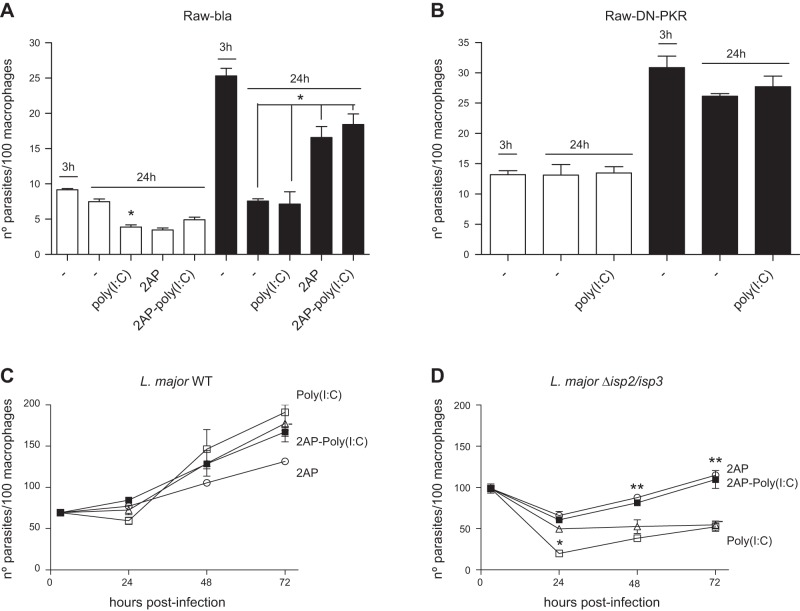
PKR activation by poly(I:C) affects survival of WT L. major in macrophages. Inhibition of serine threonine kinases promotes the survival of Δ*isp*2/*isp*3 L. major. RAW-bla (*A*) and RAW-DN-PKR (*B*) cells or macrophages from C57B6 mice were infected with WT (*C*) or Δ*isp*2/*isp*3 L. major (*D*) for 3 h, washed, and fixed (3 h) or further cultured (24–72 h). Infected cells were treated with 2AP (2 mM) for 1 h immediately after the 3 h infection or with poly(I:C) (25 μg/ml) overnight. Where indicated, the infected cells were treated with 2AP followed by poly(I:C). Open bars: WT; solid bars: Δ*isp2/3*. Experiments were performed 3 independent times. Graphs are representative of 1 experiment and indicate means ± sd. *P < 0.01, **P < 0.001.

The consequences of PKR activation were then evaluated by direct manipulation of PKR activity with poly(I:C) or by its inactivation through the kinase inhibitor 2AP (**Fig. 5**). First, we observed that WT L. major is sensitive to PKR activation, since the survival of intracellular parasites was reduced by half after incubation with poly(I:C) in both RAW (Fig. 5*A*, open bars) and primary (Fig. 5*C*, triangles) macrophages. Poly(I:C) treatment had no effect in the survival of WT parasites in the RAW-DN-PKR cells (Fig. 5*B*, open bars), confirming its relation to PKR activation. It did not promote further killing of Δ*isp*2/*isp*3 in the RAW cells, supporting the proposal that during macrophage infection, Δ*isp*2/*isp*3 induces PKR activation above threshold levels (Fig. 5*A*, solid bars). This hypothesis is further supported by the observation that the 2AP inhibitor enhanced Δ*isp*2/*isp*3 survival (Fig. 5*A*, solid bars). However, poly(I:C) increased the elimination of intracellular Δ*isp*2/*isp*3 by peritoneal macrophages 24 h after infection (Fig. 5*D*, squares). This phenomenon could be a consequence of different thresholds for the activation of PKR in RAW *vs.* peritoneal macrophages. Poly(I:C) had no effect in infected primary macrophages in the presence of 2AP, confirming that its action depends on kinase activity.

**Figure 6.**
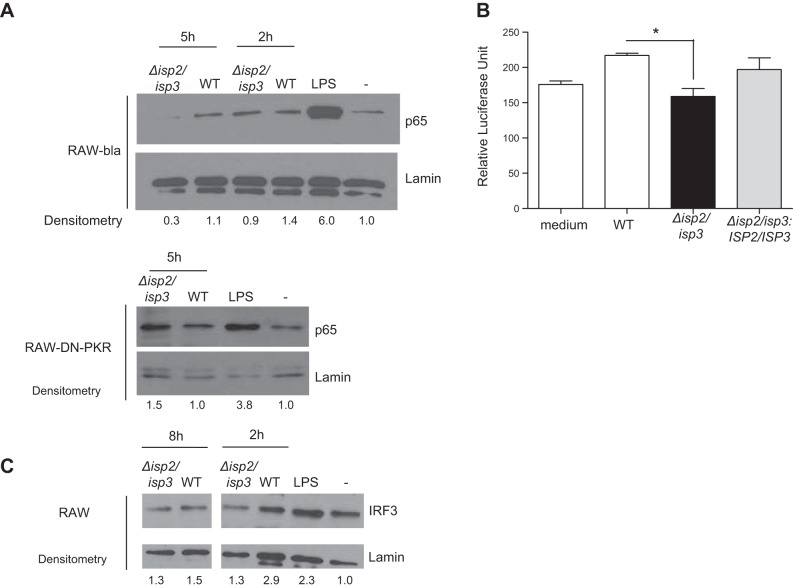
ISP2 is necessary for sustained nuclear NF-κB in macrophages infected with L. major. *A*, *C*) Cells were infected for 2, 5, or 8 h, and the nuclear extracts were processed for Western blot analysis with antibodies to NF-κB subunit p65 (*A*), IRF3 (*C*), or nuclear lamin, as the loading control. *B*) NF-κB promoter activation. RAW cells were transiently transfected with the reporter plasmid p6Kb-LUC, which contains 6 consensus-binding sites for NF-κB, located upstream of the luciferase gene, and were infected for 24 h. Noninfected cells were used as negative controls for basal luciferase activity. Experiments were performed 2 independent times. Open bars: WT; solid bars: Δ*isp2/isp3*; gray bars: Δ*isp2/isp3:ISP2-ISP3*. *P < 0.05.

PKR activation by poly(I:C) can induce the activation of the NF-κB p65/p50 dimer that, in turn, provokes the synthesis of iNOS. However, PKR activation promoted by L. amazonensis in macrophages, but not by L. major, induces the NF-κB p50/p50 repression homodimer (11, 16). We asked whether PKR activation by Δ*isp*2/*isp*3 modifies the balance of transcription factors (**Fig. 6**). We detected intact p65 in the nuclear fraction of RAW-bla cells 2 h after infection with WT L. major (Fig. 6*A*). However, the amounts of nuclear p65 were reduced to control levels in the cells infected with Δ*isp*2/*isp*3. Intact p65 diminished with time (5 h), suggesting that, as observed with L. amazonensis, infections with Δ*isp*2/*isp*3 might lead to cleavage of p65. Notably, the levels of p65 were nearly identical in the RAW-DN-PKR cells infected with either WT or Δ*isp*2/*isp*3 after 5 h. The levels of the p50 subunit were unchanged in the RAW or RAW-DN-PKR cells infected with either WT or Δ*isp*2/*isp*3 L. major (data not shown). A reporter assay was used to evaluate NF-κB-dependent promoter activation, showing a 28% increase in the expression of luciferase in RAW cells infected with WT L. major, in relation to resting cells (Fig. 6*B*). This effect was not observed in cells infected with Δ*isp*2/*isp*3 and was restored in those infected with the complemented parasite line Δ*isp*2/*isp*3:*ISP2/ISP3* (Fig. 6*B*). Levels of IRF3 were likewise reduced in the RAW cells infected with Δ*isp*2/*isp*3 for 2 h, in relation to WT L. major, and no increase was observed up to 8 h (Fig. 6*C*).

**Figure 7.**
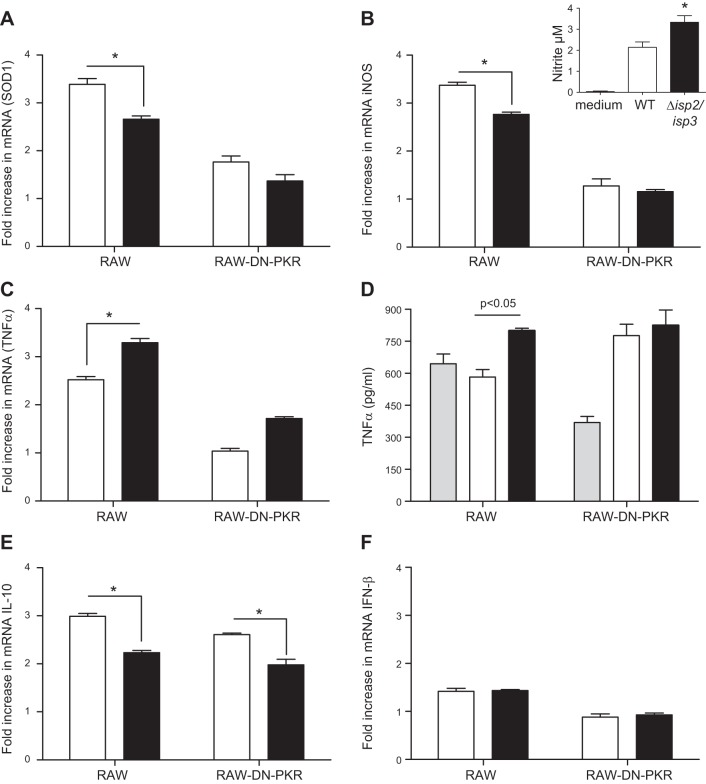
Alterations in gene expression of macrophages infected with Δ*isp*2/*isp*3 L. major are dependent on PKR. Cells were infected for 4 h, and cDNAs were used as templates in qPCR assays. Experiments were performed 3 independent times in duplicate. Graphs represent mean ± sd fold increase in SOD1 (*A*), iNOS (*B*), TNFα (*C, D*), IL-10 (*E*), and IFNβ (*F*) in relation to uninfected cells (set as 1.0) in the 3 experiments. Inset: RAW cells were incubated with recombinant IFNγ (100 ng/ml) for 3 h and infected for 18 h. Nitrite concentration was evaluated in parasite-free supernatants. Experiments were performed 2 independent times. In *D*, cells were incubated with recombinant IFNγ (100 ng/ml) for 3 h before infection, and the supernatants were tested by ELISA. experiments were performed 3 independent times. Graphs are representative of 1 experiment. Open bars: WT; solid bars: Δ*isp2/isp3*; gray bars, uninfected cells. Statistical analyses were performed with 2-way ANOVA. *P < 0.05.

To further address changes in gene expression, we performed qPCR for selected genes involved in the control of Leishmania infection (**Fig. 7**). First, we observed a 3-fold increase in mRNA of SOD1 (Fig. 7*A*) and iNOS (Fig. 7*B*) in the RAW-bla cells, but not in the RAW-DN-PKR cells infected with WT L. major (open bars) that was significantly higher than that in cells infected with Δ*isp*2/*isp*3 (solid bars) in 4 h exposures. However, Western blot analyses showed that iNOS increased transiently after 2 h in the RAW cells infected with Δ*isp*2/*isp*3 (Supplemental Fig. S2), decaying by 4 h. There was increased production of nitrite in cells infected with Δ*isp*2/*isp*3, compared with those infected with WT (Fig. 7*B*, inset). The discrepancy between RT-PCR for iNOS and NO production may be related to the transient iNOS elevation and the priming with INFγ before infection. There was a significant increase in TNF-α mRNA, which was more prominent in the RAW cells infected with Δ*isp*2/*isp*3 and was reduced in the RAW-DN-PKR cells (Fig. 7*C*). The extracellular levels of TNF-α were above those of uninfected cells only in RAW-bla infected with Δ*isp*2/*isp*3 (Fig. 7*D*). Contrary to response with L. amazonensis, the RAW or RAW-DN-PKR cells infected with Δ*isp*2/*isp*3 showed decreased levels of mRNA of IL-10, compared to cells infected with WT parasites, being independent of PKR (Fig. 7*E*). Both WT and Δ*isp*2/*isp*3 promoted IFNβ expression to the same extent, albeit at very low levels (1.5-fold; Fig. 7*F*).

**Figure 8.**
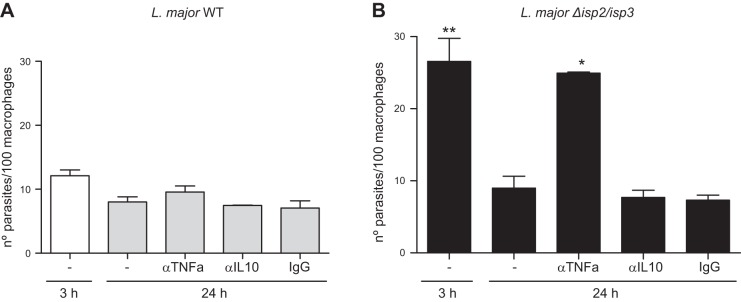
TNF-α is necessary for the killing of intracellular Δ*isp*2/*isp*3. RAW-Bla (*A*) or RAW DN-PKR (*B*) cells were infected for 3 h, washed, and cultured overnight (24 h). Neutralizing antibodies to TNF-α, IL-10, or control rat IgG were added at 10 μg/ml to the cultures immediately after the 3 h infection and left overnight. Experiments were performed 2 independent times in triplicate. Graphs are representative of 1 experiment and indicate means ± sd experiments performed in triplicate. *P < 0.01, **P < 0.001.

Given that there was an inverse correlation in the induction of TNF-α and IL-10 by Δ*isp*2/*isp*3, we asked whether increased TNF-α levels could contribute to the elimination of intracellular Δ*isp*2/*isp*3 (**Fig. 8**). The number of intracellular WT L. major parasites was unchanged 24 h after infection of the RAW cells and was unaffected by treatment with antibodies to TNF-α or IL-10 (Fig. 8*A*). In contrast, the elimination of Δ*isp*2/*isp*3 in 24 h was prevented in the presence of antibodies to TNF-α, but not in antibodies to IL-10 or control IgG (Fig. 8*B*), indicating that this cytokine largely contributes to parasite death in macrophages.

**Figure 9.**
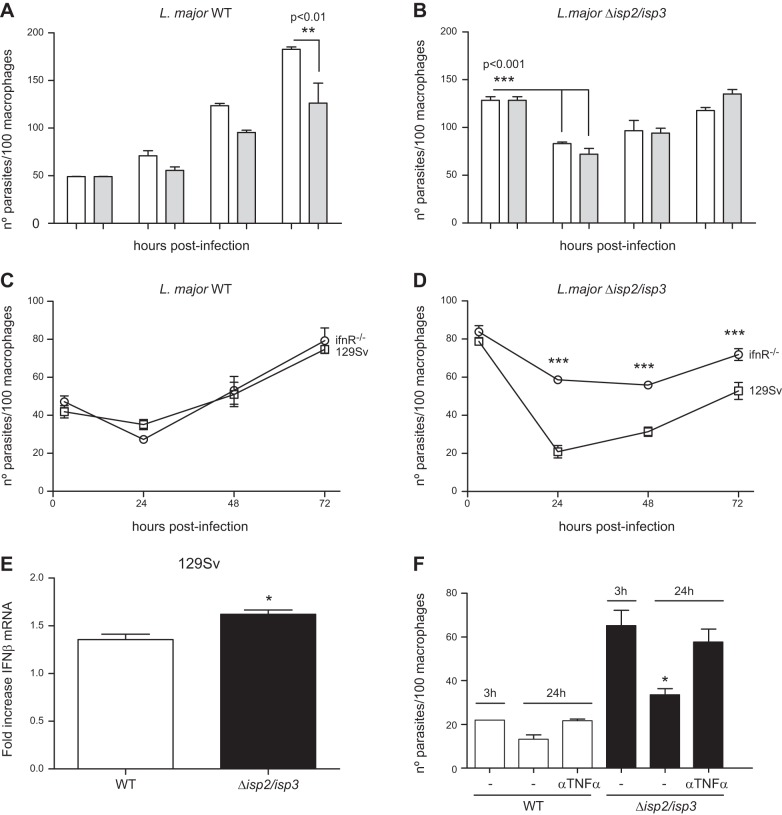
Induction of IFNβ and TNF-α in primary macrophages limits the intracellular growth of Δ*isp*2/*isp*3. Macrophages from C57B6 (*A*, *B*), 129Sv (*C*, *E*), and *ifnR*^−/−^ mice (*D*) were infected for 3 h, washed, and fixed or cultured for an additional 24–72 h. Recombinant IFNβ (1000 U/ml; *A*, *B*) or antibodies to TNF-α (*F*) were added to the cultures immediately after the 3 h infection. Experiments were performed 3 independent times. The figure shows a representative graph of 1 experiment. *E*) Macrophages from 129Sv mice were infected for 4 h, and the cDNAs were used as templates in qPCR assays. Graphs represent mean ± sd fold increase in relation to uninfected cells (set as 1.0) and are representative of 2 independent experiments. Statistical analyses were performed with 2-way ANOVA. *P < 0.01, ***P < 0.001 *vs*. all other time points.

**Figure 10.**
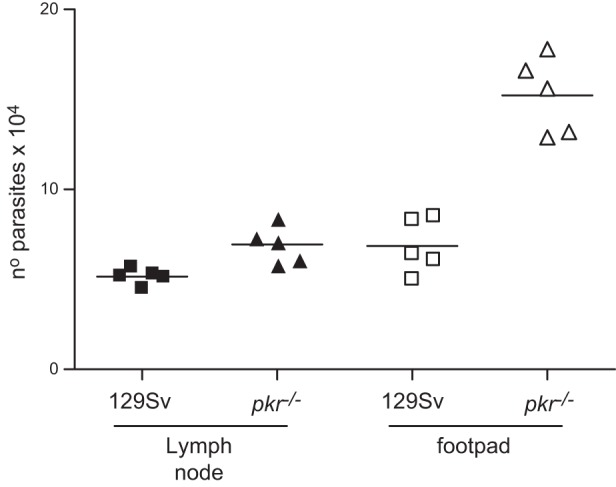
PKR controls *in vivo* infection by Δ*isp*2/*isp*3 L. major. Footpads of 129Sv or *pkr*^−/−^ mice were inoculated with 2 × 10^5^ purified metacyclic L. major Δ*isp*2/*isp*3. After 48 h, the parasite load was assessed by limiting dilution and culture for 5–7 d at 25°C. Experiment was performed 3 independent times (*n*=5/group).

**Figure 11.**
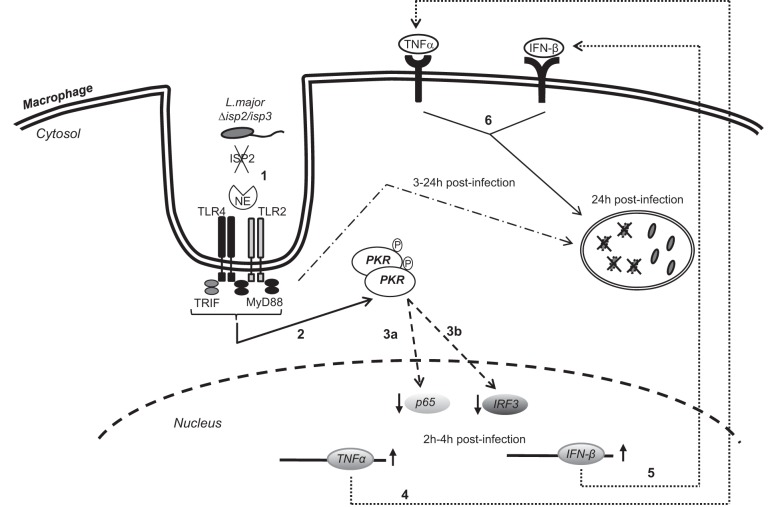
Molecular interactions in macrophages infected with L. major. Contrary to L. amazonensis, PKR activation leads to the killing of L. major, and ISP2 is a factor that prevents PKR activation. In the absence of ISP2, TLR4 and TLR2 are recruited to the site of parasite entry, and proteolytically active NE triggers those receptors (*1*), leading to the phosphorylation (*2*). The nuclear levels of the NF-κB subunit p65 (*3a*) and of interferon regulatory factor 3 (IRF3; (*3b*) are reduced, in comparison, with macrophages infected with WT L. major. The production of TNF-α (*4*) and IFNβ (*5*) is induced, which results in parasite killing (*6*). Activation of TLRs *via* MyD88 and TRIF is necessary for the killing of ∼50% of the internalized parasites within 24 h.

The consequences of IFNβ production in response to intracellular L. major were then evaluated in primary macrophages (**Fig. 9**). Treatment with IFNβ reduced the growth in macrophages of WT L. major after 2–3 d (Fig. 9*A*), whereas the elimination of Δ*isp*2/*isp*3 after 24 h and the reduced growth rate that followed were unaffected by IFNβ treatment (Fig. 9*B*). It is possible that the primary macrophages infected with Δ*isp*2/*isp*3 secreted amounts of IFNβ that were sufficient to cause parasite death, being thus refractory to exogenously added IFNβ. To test this possibility, we followed the kinetics of parasite growth in macrophages deficient in type 1 IFN receptor (IFNR), or from the background mouse strain. WT L. major grew normally in either macrophage type (Fig. 9*C*), whereas the growth of Δ*isp*2/*isp*3 was significantly increased in *IFNr*^−/−^ mouse macrophages (Fig. 9*D*), suggesting that IFNβ contributes to the poor intracellular development of Δ*isp*2/*isp*3. IFNβ expression in macrophages infected with Δ*isp*2/*isp*3 was significantly higher than in those infected by WT (Fig. 9*E*). In addition, neutralizing antibodies to TNF-α prevented the death of Δ*isp*2/*isp*3, providing evidence that endogenous TNF-α plays a role in controlling parasite survival (Fig. 9*F*). Finally, we evaluated the relative contribution of PKR to short-term mouse infections, to avoid the potential interference of the adaptive immune response. The parasite load of Δ*isp*2/*isp*3 at the lymph nodes 48 h after infection was similar in the 129Sv and *pkr*^−/−^ mice, whereas the number of parasites at the site of infection (footpad) was 2-fold higher in the *pkr*^−/−^ mice than in the 129Sv mice (**Fig. 10**). These results are in agreement with the *in vitro* observations that, in the absence of active PKR, Δ*isp*2/*isp*3 survive and multiply inside macrophages and set PKR as a negative regulator of L. major growth, when active NE and TLR4 are mobilized during parasite uptake (**Fig. 11**).

## DISCUSSION

PKR activation has been associated with the antiviral and antiproliferative effects of IFNs for many years (17). Macrophages infected with L. amazonensis activate PKR and sustain IFNβ expression, which boosts intracellular parasite growth (12). We report results from our study showing that PKR activation is not necessary for L. major intracellular growth in macrophages and is prevented by parasite ISP2. In addition, when PKR is activated by exogenous poly(I:C) it provokes partial parasite elimination, revealing that, contrary to the effect on L. amazonensis, PKR activation is detrimental to the intracellular development of L. major.

Bacterial LPS can activate PKR *via* TLR4, involving a physical association between the adaptor proteins MyD88 and PKR (18). L. major ISP2 prevents the activation of TLR4 (10), and we showed in our study that the increased phagocytosis followed by killing of the L. major knockout lines Δ*isp*2/*isp*3 depended on PKR, most likely because of the triggering of TLR4-TLR2-NE. The use of an inhibitor to NE linked PKR activation to NE activity. Even though the synthetic inhibitor that we used may also target proteinase 3, its expression has not been reported in macrophages, to our knowledge. Furthermore, experiments using neutralizing antibodies linked CD11b (a subunit of the CR3 receptor), TLR4, TLR2, and NE activities to the PKR pathway downstream.

ISP2 is expressed by L. amazonensis, albeit at lower levels than in L. major (not shown) and may be available to prevent activation of the PKR pathway. However, PKR activation by L. amazonensis was associated mainly with TLR2 *via* parasite LPG (12). It is possible that variations in the surface LPG of Leishmania species affect the levels of TLR2 engagement, with influence on the control of PKR activation and expression. In addition, colocalization between TLR2 and WT L. major at the site of entry in macrophages was not observed. In contrast, the involvement of TLR2 and TLR4 in the infection by Δ*isp*2/*isp*3 was further supported by colocalization. This is the first time that a close association between TLR2, TLR4, and the parasite has been observed in Leishmania-infected macrophages. It is well known that TLR2 activation correlates with protection against L. major, which is consistent with our observation that TLR2 contributes to the killing of Δ*isp*2/*isp*3, since Δ*isp*2/*isp*3 survived in macrophages from TLR2-deficient mice. This observation links the inhibitory activity of ISP2 to an important step in preventing the engagement, not only of TLR4 but also of TLR2, contributing to successful infection. In agreement with that observation, MyD88 and TRIF were essential for parasite killing within 24 h. A plausible explanation is that CR3, TLR4, and TLR2, which are segregated in resting macrophages, are recruited to a single signaling platform, by as yet unknown factors, on infection with L. major (Fig. 11). In WT parasites, ISP2 blocks NE activity, preventing engagement of such a signaling platform during parasite phagocytosis and ensuring subsequent survival. However, it remains to be determined whether, once in the parasitophorous vacuole, the receptors continue to convey cellular responses at later times.

The modulation of NF-κB subunit dimerization and of its steady-state levels is one of the main mechanisms responsible for the subversion of cytokine profile expression and macrophage activation by Leishmania parasites. Relevant to this study, in THP-1-differentiated macrophages, L. amazonensis induced the cleavage of the p65 subunit in a PKR-dependent fashion, whereas L. major was found to preserve the p65/p50 dimer (11). There was a significant reduction in the levels of p65 in cells infected with Δ*isp*2/*isp*3 after 5 h, which was linked to PKR and can ultimately influence the control of gene expression. It is known that LPS-induced TLR4 activation triggers NF-κB in the first few hours, and its sustained activation depends on the maintenance of feed-forward mechanisms by autocrine stimuli and on chromatin remodelling (19). It is possible that such mechanisms are not engaged by Δ*isp*2/*isp*3, leading to the reduction of nuclear p65 after 5 h, either by degradation or nuclear export. We did not observe lower molecular mass fragments of p65 (data not shown) suggesting that infection with Δ*isp*2/*isp*3 may promote its full degradation. At least with L. mexicana, promastigotes cleave the p65 subunit, generating a smaller p35 subunit, whereas amastigotes fully degrade the p65 subunit (20, 21). However, in L. mexicana, degradation of p65 was related to the parasite cathepsin L-like cysteine peptidase (CPB), which should not be altered by deletion of ISPs in L. major. The absence of NF-κB promoter activation by Δ*isp*2/*isp*3 in luciferase-reporter assays is consistent with low levels of nuclear NF-κB. Indeed, in thioglycolate-recruited macrophages infected with L. major, very little NF-κB was available for binding to its respective DNA elements (22).

We detected changes in the mRNA levels for a few genes in L. major-infected RAW cells, and of relevance, there was PKR-dependent increased expression of TNF-α on infection with Δ*isp*2/*isp*3. Neutralizing antibodies provided evidence that this cytokine is associated with the killing of intracellular Δ*isp*2/*isp*3, which is consistent with the finding that TNF-α is leishmanicidal in macrophages that had been infected with L. major and subsequently exposed to apoptotic neutrophils (23). Our results suggest that the NE-TLR4 pathway operating in macrophages during parasite uptake can likewise induce TNF-dependent leishmanicidal activity in the absence of neutrophils. The basal levels of TNF-α in noninfected cells were elevated, possibly by the priming with IFNγ used in our *in vitro* model, and we cannot exclude that potential greater variations in the TNF levels are present *in vivo*. The down-regulation of SOD1 expression could also be relevant and fits well with our previous data showing that ROS largely contribute to the killing of Δ*isp*2/*isp*3 in macrophages of C57B6 mice (10). The increase in the burden of L. amazonensis and L. braziliensis in macrophages provoked by IFNβ relies on the enhancement of SOD1 levels that reduce superoxide release (24). Even though the main leishmanicidal effect of IFNβ on L. major-infected macrophages relates to increases in iNOS, it is possible that the induction of SOD1 by PKR helps to counterbalance the negative effect of IFNβ and iNOS, contributing to infection.

Finally, we found important differences in the susceptibility of WT and Δ*isp*2/*isp*3 to endogenous IFNβ. The association of type 1 IFNs with Leishmania infections has been well studied. A role for IFNα/β in the protection against L. major *in vivo* was found to involve the induction of iNOS (25). *In vitro*, the infection of macrophages with L. major induces the release of type 1 IFNs, which is essential for the expression of iNOS and leishmanicidal activity. In contrast, a protective effect is exerted when high amounts of IFNβ are added to macrophages 2 h before infection, by inhibition of the synthesis of NO, leading to NF-κB degradation (22). We detected very little increase in IFNβ expression in the L. major-infected-RAW cells (1.5-fold) as compared to that observed in the peritoneal macrophages infected by L. amazonensis (300-fold; 12), and the small increase was dependent on PKR for both WT and Δ*isp*2/*isp*3. This result is in agreement with the observations that IFNβ promotes the killing of L. major in macrophages, whereas it favors the proliferation of L. amazonensis. In contrast, IFNβ production induced by LPS, for example, does not require PKR (26), illustrating that TLR4-induced IFNβ may involve multiple pathways.

IFNβ treatment protects BALB/c mice from infections with L. major in a dose-dependent manner (27), supporting the view that the sensitivity of L. major to type 1 IFNs is associated with local levels and the kinetics of macrophage exposure. WT L. major grew normally in macrophages of 129Sv and IFNR-knockout mice, suggesting that the autocrine pathways involving type 1 IFN are absent or largely attenuated in this model. However, IFNβ added exogenously to 129Sv-derived infected macrophages impaired the growth of WT L. major, at least to some extent, but not of Δ*isp*2/*isp*3. It is possible that, in the absence of ISPs, IFNβ levels produced by those macrophages are sufficient to promote parasite death, precluding the observation of further parasite killing by exogenous IFNβ. Indeed, IFNβ-associated pathways seem to be fully operational in 129Sv, as the intracellular growth of Δ*isp*2/*isp*3 was rescued in macrophages from IFNR-knockout mice and confirm that in the absence of ISPs, triggering of the leishmanicidal machinery by L. major depends on IFNβ. The endocytosis of TLR4 induces the production of IFNβ in a pathway dependent on TRAM (28). We observed colocalization between TLR4 and Δ*isp*2/*isp*3 during macrophage infection, suggesting that it is internalized together with the parasite, which could trigger IFNβ production.

In summary, we propose that ISP2 helps to control the induction of type-1 IFNs and of TNF-α by L. major by minimizing the PKR pathway and is an important adaptation for parasite survival in the host.

## Supplementary Material

Supplemental Data
