# Supplementary material for: Role of protein kinase R in the killing of Leishmania major by macrophages in response to neutrophil elastase and TLR4 via TNFα and IFNβ
Source: FASEB J. 2014 Jul;28(7):3050–63. doi: 10.1096/fj.13-245126 (PMC4210457; doi:10.1096/fj.13-245126)
Supplement: Supplemental Data [file supp_28_7_3050__index.html]

Role of protein kinase R in the killing of Leishmania major by macrophages in response to neutrophil elastase and TLR4 via TNFα and IFNβ — Role of protein kinase R in the killing of Leishmania major by macrophages in response to neutrophil elastase and TLR4 via TNFα and IFNβ — Supplemental Data 

# Role of protein kinase R in the killing of Leishmania major by macrophages in response to neutrophil elastase and TLR4 v*ia* TNFα and IFNβ

## Supplemental Data

**Files in this Data Supplement:**

- Supplemental Data - (*13-245126SuppData.zip; compressed file 273 KB*)
