# Supplementary material for: Role of protein kinase R in the killing of Leishmania major by macrophages in response to neutrophil elastase and TLR4 via TNFα and IFNβ
Source: FASEB J. 2014 Jul;28(7):3050–63. doi: 10.1096/fj.13-245126 (PMC4210457; doi:10.1096/fj.13-245126)
Supplement: Supplemental Data [file supp_fj.13-245126_13-245126SuppData.zip › Supplemtal Fig 1.pdf]

Supplemental Figure I

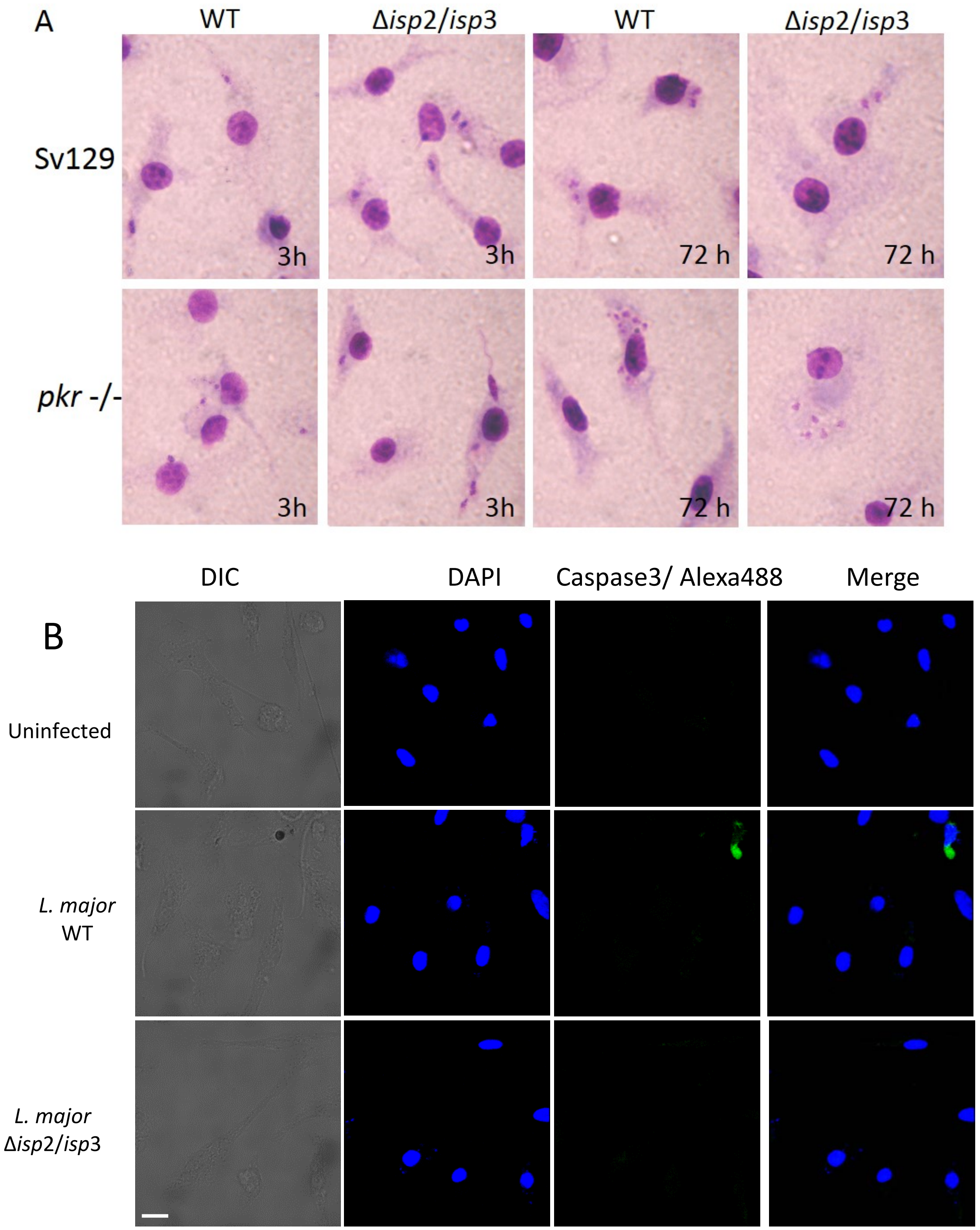

Supplemental Fig1: Primary macrophages infected with *L. major*. Thioglycolate-elicited macrophages of Sv129 (A) or C57B6 (B) mice were infected and cultured for the indicated times. Cells were giemsa stained (A) or processed for immunofluorescence using anti-activated caspase 3 (green). Samples were analysed by confocal microscopy. Scale bar: 5 µm.
