# Supplementary material for: Role of protein kinase R in the killing of Leishmania major by macrophages in response to neutrophil elastase and TLR4 via TNFα and IFNβ
Source: FASEB J. 2014 Jul;28(7):3050–63. doi: 10.1096/fj.13-245126 (PMC4210457; doi:10.1096/fj.13-245126)
Supplement: Supplemental Data [file supp_fj.13-245126_13-245126SuppData.zip › Supplemental Fig 2n.pdf]

Supplemental Figure 2

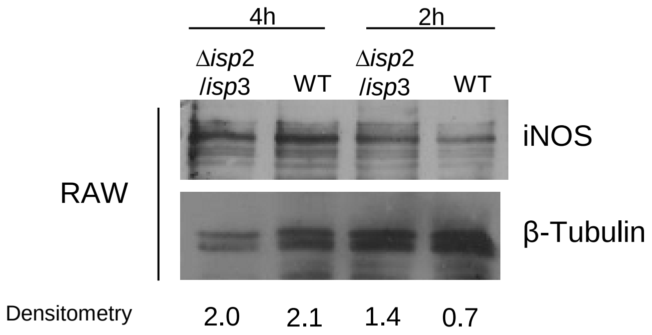

Supplemental Fig 2: Infection with Δ*isp*2/*isp*3 increase iNOS expression. Cells were infected for 2 h or 4h, washed and lysed. Extracts were processed for Western blot with antibodies to iNOS or β-tubulin as a loading control. The numbers indicate the relative levels of iNOS normalized by densitometry.
